# Differences in responses to flooding by germinating seeds of two contrasting rice cultivars and two species of economically important grass weeds

**DOI:** 10.1093/aobpla/plu064

**Published:** 2014-10-20

**Authors:** Lucy P. Estioko, Berta Miro, Aurora M. Baltazar, Florinia E. Merca, Abdelbagi M. Ismail, David E. Johnson

**Affiliations:** 1 Bicol University, Legazpi City, Albay, Philippines; 2 International Rice Research Institute, Los Banos, Laguna, Philippines; 3 University of the Philippines Los Banos, College, Laguna, Philippines

**Keywords:** Alcohol dehydrogenase, aldehyde dehydrogenase, anaerobic germination, barnyard grass, direct-seeded rice, *Echinochloa colona*, *Echinochloa crus-galli*, fermentative metabolism, pyruvate decarboxylase, rice weeds.

## Abstract

Crop productivity is largely affected by abiotic factors such as flooding and by biotic factors such as weeds. Although flooding after direct seeding of rice helps suppress weeds, it also can adversely affects germination and growth of rice, resulting in poor crop establishment. Barnyard grasses (*Echinochloa* spp.) are among the most widespread weeds affecting rice, especially under direct seeding. The present work aimed to establish effective management options to control these weeds. We assessed the effects of variable depths and time of submergence on germination, seedling growth and carbohydrate metabolism of (i) two cultivars of rice known to differ in their tolerance to flooding during germination and (ii) two barnyard grasses (*Echinochloa colona* and *E. crus-galli*) that commonly infest rice fields. Flooding barnyard grasses with 100-mm-deep water immediately after seeding was effective in suppressing germination and growth. *Echinochloa colona* showed greater reductions in emergence, shoot and root growth than *E. crus-galli*. Delaying flooding for 2 or 4 days was less injurious to both species. *Echinochloa colona* was also more susceptible to flooding than the flood-sensitive rice cultivar ‘IR42’. The activity of alcohol dehydrogenase (ADH) and pyruvate decarboxylase (PDC) in rice seedlings was increased by flooding after sowing but with greater increases in ‘Khao Hlan On’ compared with ‘IR42’. The activity of ADH and PDC was enhanced to a similar extent in both barnyard grasses. Under aerobic conditions, the activity of ADH and PDC in the two barnyard grasses was downregulated, which might contribute to their inherently faster growth compared with rice. Aldehyde dehydrogenase activity was significantly enhanced in flood-tolerant ‘Khao Hlan On’ and *E. crus-galli*, but did not increase in flood-sensitive *E. colona* and ‘IR42’, implying a greater ability of the flood-tolerant types to detoxify acetaldehyde generated during anaerobic fermentation. Confirmation of this hypothesis is now being sought.

## Abstract (precis)

Barnyard grasses are serious weeds in direct seeded rice. We assessed the effectiveness of using controlled flooding for its control using two rice cultivars and two barnyard grasses contrasting in flood tolerance during germination. Flooding with 100 mm water after seeding suppressed barnyard grasses; but delaying flooding by 2-4 days was ineffective. Flooding increased the activity of alcohol dehydrogenase and pyruvate decarboxylase; the increase was higher in the tolerant rice cultivar but similar in both barnyard grasses. Aldehyde dehydrogenase activity increased only in flood-tolerant types of rice and weeds, but not in flood-sensitive types*,* implying potential role in tolerance.

## Introduction

Abiotic stresses such as flooding and biotic factors such as weed infestation cause considerable reductions in crop productivity worldwide. Flooding the soil during crop establishment can help control most weeds and reduce production cost. But this is practical only if the crop can tolerate flooding during germination and early growth. The present paper examines the physiological aspects of this approach for controlling weeds in the rice crop. In response to water and labour shortages and for other benefits (Ismail *et al.* 2012) Asia's rice farmers are progressively shifting from transplanting to direct seeding. However, weed competition in direct-seeded fields can be intense since both weeds and rice germinate together. Consequently, yield losses due to weed competition have been reported to be 3-fold greater in direct-seeded rice than in transplanted rice (Hill *et al.* 1990).

In direct-seeded rice, controlled flooding of the field is usually delayed until rice seedlings have emerged. Unfortunately, this also brings about the simultaneous emergence of certain grass weeds (Rao *et al.* 2007). It is therefore highly desirable to develop rice lines with greater tolerance to early flooding compared with the weeds. This would allow farmers to flood earlier, thereby selectively suppressing early weed growth while permitting rice seedlings to become established. Such an approach would also reduce the risk of crop losses from uncontrolled flooding events and, in some cases, poor land levelling. Previous studies have shown that rice cultivars have varied responses to flooding during germination and that certain genotypes are more tolerant of the hypoxic soil of flooded fields (Guglielminetti *et al.* 1995; Ismail *et al.* 2009; Angaji *et al.* 2010). Some of the most important and widespread weeds cohabiting with rice are *Echinochloa* spp. (barnyard grass). Various studies showed that species such as *Echinochloa oryzoides, E. phyllopogon*, *E. crus-pavonis* and *E. crus-galli* can germinate and grow in oxygen-deficient conditions that characterize flooded soils (Pearce and Jackson 1991; Pearce *et al.* 1992; Zhang *et al.* 1994; Fox *et al.* 1995; Fukao *et al.* 2003; Chauhan and Johnson 2011; Ismail *et al.* 2012). This ability makes weed control by imposing early flooding less effective. Moreover, some echinochloa species such as *E. crus-galli* are more tolerant of flooding than others such as *E. colona* (Holm *et al.* 1977; Mujer *et al.* 1993).

Germinating rice seeds perform various growth and metabolic processes that enhance their chances of surviving oxygen deficiency. These include shifting from aerobic to anaerobic respiration. This generates less energy but the amount remains sufficient to sustain growth by the shoot of the germinating embryo (Rumpho and Kennedy 1983*a*; Guglielminetti *et al.* 1995; Miro and Ismail 2013). This implies that seeds germinating under water are able to degrade starch into soluble sugar substrates used for anaerobic respiration (Kennedy *et al.* 1980; Perata *et al.* 1996; Ismail *et al.* 2012). Key enzymes in anaerobic fermentation are pyruvate decarboxylase (PDC; E.C. 4.1.1.1), alcohol dehydrogenase (ADH; E.C. 1.1.1.1) and aldehyde dehydrogenase (ALDH, E.C. 1.2.3.1). Increases in activities of these enzymes have been linked to higher energy production, which allows faster coleoptile elongation and seedling survival (Nakazono *et al.* 2000; Stryer *et al.* 2002; Tsuji *et al.* 2003*b*; Meguro *et al.* 2006).

Understanding the growth responses to flooding in rice and weed species at early growth stages may help formulate water management strategies that exploit differential effects of flooding on rice and barnyard grasses to selectively suppress weed growth without affecting rice crop establishment. Furthermore, a better understanding of biochemical processes associated with differential sensitivity to flooding may assist in developing rice cultivars with greater tolerance of submergence during germination and early growth (Ismail *et al.* 2012). This would improve weed control and reduce the risk of poor crop establishment caused by uncontrolled floods early in the season. To date, most studies have been made of the adaptive features and responses to flooding by rice and rice weeds individually but less information is available on their comparative responses. Some earlier studies have reported contrasting responses in rice and *E. oryzoides* to poorly aerated conditions (Pearce and Jackson 1991; Pearce *et al.* 1992), where shoot elongation was enhanced in germinating rice but slowed in *E. oryzicola*.

This study aims to assess differential growth and metabolic responses in rice and barnyard grass to help develop effective management practices to control the weeds in favour of rice. We evaluated two contrasting rice cultivars and two ‘barnyard grasses’. The rice cultivar ‘IR42’ is normally high yielding but sensitive to flooding at all growth stages, while ‘Khao Hlan On’ is a traditional rice landrace with high tolerance to flooding during germination (Angaji *et al.* 2010; Ismail *et al.* 2012). *Echinochloa crus-galli* and *E. colona* are two of the most economically important grass weeds of rice with the former known to be the more tolerant of flooding in the field. We evaluated and compared germination and seedling growth and development under different flooding depths and timings in both rice and barnyard grass to assist with the development of an optimal weed management strategy through managing the timing and depth of flooding. We also evaluated changes in fermentative metabolism to help understand the response mechanisms for flooding during germination of the four genotypes.

## Methods

### Plant material

Seeds (caryopses) of rice cultivars ‘Khao Hlan On’ and ‘IR42’ were from the International Rice Research Institute, Philippines. Seeds of *E. crus-galli* and *E. colona* were collected from rice fields in Laguna Province, Philippines and multiplied from single panicles to provide common stocks. To break dormancy before sowing, seeds were incubated at 45–50 °C for up to 5 days for rice and for 5 h for barnyard grasses.

### Experimental design

Experiments were conducted in a greenhouse or incubator. Treatments were shallow flooding 5, 10 or 20 mm-deep with flooding starting 0, 2 or 4 days after seeding (DAS) or deeper flooding (100 mm) applied at 0 or 3 DAS. In both sets of experiments, a control treatment was included where the seeds or seedlings were kept at field capacity (Table 1). In all experiments, seeds of ‘Khao Hlan On’ and ‘IR42’ were sown at ∼1 cm below the soil surface, whereas the seeds of *E. crus-galli* and *E. colona* were sown at the soil surface. The soil was a mix of clay (50 %) and loam (50 %), sterilized before use. Trays were watered to field capacity in control aerobic treatments (non-flooded) or flooded by placing the trays in submergence pools with water levels ranging from 5 to 100 mm, as described (Table 1). Greenhouse yearly average conditions were: day/night cycles of 12/12 h, temperatures of 30/25 °C and humidity between 70 and 85 %. The incubator (Precision Scientific Incubator Model 818) conditions were programmed at 30/20 °C day/night temperatures, 12/12 h photoperiod and PAR of 200 µmol m^−2^ s^−1^.

**Table 1.** Experimental details including location, water depth, start of flooding, flooding duration, sampling time and the traits recorded in each experiment. Experiments were conducted across the years between 2007 and 2013.

Experiment	Location	Water depth (mm)	Flooding start (DAS)	Flooding duration/sampling time (DAS)	Measurements
Shallow flooding/waterlogging	Greenhouse	0, 5, 10, 20	0, 2, 4	21/14 and 21	% Germination Shoot and root length
Deep flooding	Incubator/greenhouse	0, 100	0	10/Daily	% Germination Shoot and root length Carbohydrate assays Enzyme activities
Late deep flooding	Greenhouse	0, 100	3	21/14 and 21	% Germination Shoot and root length

### Growth and physiological measurements

Percent germination was calculated as the ratio of germinated seeds per genotype and the number of total seeds sown. Germinated seeds were those with emerging radicle or coleoptile visible to the naked eye. Shoot and root length were measured on a glass plate (50 × 30 cm) mounted on crushed ice. Shoots included all aerial parts and roots included all below-ground material excluding seeds. At 10 DAS or less, only the coleoptile was visible in submerged seedlings. At 14 DAS and thereafter, all seedlings showed about one to three true leaves and various degrees of root development. Length measurements were recorded on the longest leaf and the longest root.

### Carbohydrate assays

Germinating seeds were carefully washed; seeds were then detached from the seedlings, frozen in liquid nitrogen and stored at −80 °C until freeze-dried and kept in a desiccator at room temperature for analysis. Dry seeds (not sown) were also analysed at Day 0 for both aerobic and flooded treatments.

Soluble sugar concentration was determined using the anthrone method as described in Fales (1951). Soluble sugars were extracted twice from 100 mg of freeze-dried tissue powder in 80 % v/v ethanol. Sugar concentration in each extract was determined colorimetrically at 620 nm using a Beckman Coulter DU 800 spectrophotometer (Brea, USA). Glucose was used as a calibration standard.

Starch concentration was determined as described by Kunst *et al.* (1988). Starch in the residue from the soluble sugar extraction was dried at 70 °C for 24 h, gelled with acetate buffer, boiled and then converted to glucose with amyloglucosidase by incubating at 37 °C for 24 h. The resulting free glucose was measured with glucose oxidase by mixing the sample with peroxidase–glucose oxidase enzyme and *o*-dianisidine dihydrochloride solution. Absorbance was measured at 450 nm.

### Enzyme assays

Seedlings were sampled every day until 10 DAS, immediately frozen in liquid nitrogen and stored at −80 °C until assayed. Dry seeds (ungerminated) were used at Day 0 in both control and flooded treatments.

#### Preparation of protein crude extract

About 500 mg of seeds or seedlings were ground to powder in liquid nitrogen and added to cold buffer composed of 100 mM HEPES, pH 7.4, 1 mM EDTA, 5 mM DTT, 0.1 % v/v, Triton X-100 and 10 % glycerol. The homogenate was centrifuged at 12 000 *g* at 4 °C for 20 min. Protein concentration was determined using Bradford reagent (Sigma B 6916) with bovine serum albumin as the standard (Bradford 1976).

#### Alcohol dehydrogenase

Total ADH activity was assayed as described by Ismail *et al.* (2009). Diluted crude extract (100 µL) was added to a reaction mixture of 51.8 mM N-[Tris(hydroxymethyl)methyl]-2-aminoethanesulfonic acid (TES), pH 7.0 and 0.17 mM NADH. Acetaldehyde (20 mM) was added to start the reaction towards ethanol synthesis and ADH activity was monitored by oxidation of NADH. Samples were read at 340 nm under ambient conditions (25 °C) for 180 s.

#### Pyruvate decarboxylase

Total PDC activity was analysed using the procedures described by Quimio *et al.* (2000). Crude extract was added to a mixture containing 1 % bovine serum albumin, 41.67 mM 2-(N-Morpholino)ethanesulfonic acid (MES) and 0.5 mM thiamine pyrophosphate (TPP) and then centrifuged at 10 000 *g* at 4 °C for 3 min. TES buffer pH 8.0 was added to the supernatant to a final concentration of 446 mM and incubated at 25 °C for 1 h. For the activity assay, the resulting solution (100 µL) was added to a reaction mixture containing 62.5 mM MES, 0.5 mM TPP, 50 mM oxamate, 10 U ADH and 0.17 mM NADH. Sodium pyruvate (10 mM) was added to initiate the reaction, wherein acetaldehyde produced through the action of PDC was concomitantly reduced by ADH. Simultaneous oxidation of NADH was used to monitor PDC activity. Total volume for each of the reaction mixtures was 1000 µL. Samples were spectrophotometrically read at 340 nm under ambient conditions.

#### Aldehyde dehydrogenase

Aldehyde dehydrogenase activity was assayed according to Fukao *et al.* (2003). Crude protein extract (100 µL) was added to a reaction mixture containing 100 mM sodium pyrophosphate (pH 9.5) and 1.5 mM NAD^+^. Acetaldehyde (0.34 mM) was added to start the reaction towards its oxidation and the accompanying reduction of NAD^+^ was used to monitor the activity of ALDH. Total volume of the reaction mixture was 1000 µL. Samples were read at 340 nm under ambient conditions.

### Immunodetection of ALDH2a and ALDH2b

Thirty micrograms of total protein was loaded per sample in 8 % sodium dodecyl sulphate–polyacrylamide gel electrophoresis and subjected to electrophoresis (Laemmli 1970) prior to in-gel detection. The antibody (courtesy of Dr M. Nakazono) was a rabbit monoclonal antibody against ALDH2 synthesized from *Oryza sativa* and *Arabidopsis thaliana* peptides (Nakazono *et al.* 2000), detecting both ALDH2a and ALDH2b. The signal was developed by chemiluminescence following manufacturer's instructions (Novex ECL HRP kit, Invitrogen, USA).

### Statistical design and analysis

Experiments in the greenhouse were replicated four times, while those in the incubator were replicated three times. Treatments in all studies were arranged in a randomized split-plot design with flooding depth as the main plot and timing of flooding as the subplot. Analyses of variance were performed using CropStat for Windows (Version 6.1, 2007) and treatment means were compared using LSD (*P* < 0.05).

## Results

### Germination and growth responses to different flooding depths and times

Three flooding regimes were used to assess differential responses of rice and barnyard grass during germination under submergence, as well as to evaluate the effectiveness of flooding as a weed control measure under different direct-seeding scenarios. The first two experiments compared different water depths. The first experiment imposed shallow flooding (5, 10 or 20 mm deep) applied at 0, 2 and 4 DAS. The second involved deeper flooding (100 mm) applied at seeding. The third experiment investigated the effect of late flooding (starting 3 DAS) compared with the early flooding (starting 0 DAS).

**Figure 1.**
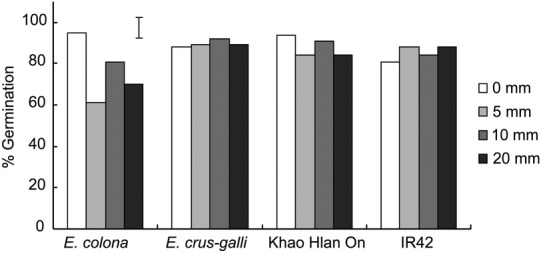
Percent germination of *E. crus-galli*, *E. colona*, ‘Khao Hlan On’ and ‘IR42’ submerged in 0, 5, 10 and 20 mm of water immediately after seeding. Vertical bars indicate l.s.d. at *P* < 0.05.

Shallow flooding to 5–20 mm beginning immediately after seeding (0 DAS) did not significantly reduce percent germination of rice cultivars ‘IR42’ and ‘Khao Hlan On’ or of *E. crus-galli*. However, in flooding-intolerant *E. colona*, germination was decreased when flooded to 5 mm (Fig. 1). This reduction was not statistically significant under 10 mm flooding. Delaying flooding to 2 DAS or to 4 DAS had no effect on germination since it had already commenced in all genotypes.

While shallow flooding up to 20 mm did not prevent germination by any of the lines tested, it reduced shoot lengths of *E. colona*, *E. crus-galli* and ‘IR42’ (Table 2, **see Supporting Information**) 7 and 14 DAS. In the case of ‘Khao Hlan On’, shoot elongation was unaffected by 5 and 10 mm flooding although 20-mm flooding was inhibitory. The setback to shoot elongation resulting from flooding treatment starting at 0 DAS was recovered strongly by rice. The shorter shoots, evident at 7 and 14 DAS, compared with controls were no longer statistically shorter by 21 DAS in ‘Khao Hlan On’ or ‘IR42’ (‘Khao Hlan On’ was the tallest genotype). In contrast, shoot lengths remained significantly shorter in *E. crus-galli* and *E. colona* (Table 2) throughout when flooded at 0 DAS. When flooding was delayed from 0 to 2 DAS, shoot lengths measured at 14 and 21 DAS were significantly reduced in both barnyard grasses (Table 2; **see Supporting Information**) but not in the rice cultivars. When flooding was started at 4 DAS it had no significant effects on shoot lengths of either of the two rice cultivars or of the barnyard grasses, even after 21 days of flooding (Table 2).

**Table 2.** Comparative lengths of the shoots (mm) of *E. colona*, *E. crus*-galli, ‘Khao Hlan On’ and ‘IR42’ at different flooding depths and timing. Seeds were sown in soil and flooded to depths of 5, 10 and 20 mm. Controls were sown under aerobic conditions (0 mm). Flooding was started at 0, 2 or 4 DAS and finished 7, 14 or 21 DAS. Bold numbers with (*) indicate values are significantly different from the control at LSD_0.05_.

Flooding treatment		7 DAS				14 DAS				21 DAS			
Start	Depth (mm)	*E. colona*	*E. crus-galli*	‘IR42’	‘Khao Hlan On’	*E. colona*	*E. crus-galli*	‘IR42’	‘Khao Hlan On’	*E. colona*	*E. crus-galli*	‘IR42’	‘Khao Hlan On’
**0 DAS**	0	43	84	131	138	256	316	308	390	435	470	389	526
	5	30	55*	83*	122	173*	242*	266*	376	364*	420*	392	518
	10	29	54*	76*	117	170*	231*	254*	368	391*	419*	399	531
	20	29	44*	68*	84*	149*	211*	236*	341*	361*	387*	404	500
				LSD_0.05_	*27*			LSD_0.05_	*25*			LSD_0.05_	*48*
**2 DAS**	0	42	84	129	154	259	319	305	378	432	485	377	521
	5	43	72	128	143	232*	285*	292	392	427	481	392	540
	10	45	67	131	151	227*	270*	297	380	436	451*	388	531
	20	42	66	120	140	189*	255*	283	384	396*	444*	408	544
				LSD_0.05_	*25*			LSD_0.05_	*25*			LSD_0.05_	*29*
**4 DAS**	0	42	90	130	155	254	320	299	406	449	489	393	546
	5	46	86	130	151	255	324	312	409	449	485	404	543
	10	51	84	136	155	253	319	306	404	449	482	380	533
	20	48	88	139	161	236	309	307	406	428	474	417	546
		* *	* *	LSD_0.05_	*26*			LSD_0.05_	*25*	* *	* *	LSD_0.05_	*37*

Early flooding, starting on the day of sowing, reduced root length strongly in *E. colona* and rice when measured at 7 DAS and in all genotypes when measured at 14 DAS. However, rice roots recovered their length to that of the controls at 21 DAS. Similar effects were noticed when flooding began at 2 DAS, while the effects were less distinct when flooding was delayed until 4 DAS (Table 3). Sensitive genotypes were most affected by flooding depth and timing, with root length reductions of up to 50 % in ‘IR42’ and 34 % in *E. colona* at 7 DAS when 20 mm water depth was applied at seeding (Table 3). Apparently, flooding decreased shoot and root lengths compared with aerobic conditions. Although flooding starting at 4 DAS did not significantly affect shoot elongation of any genotype (Table 2) it significantly reduced root length in both rice genotypes by 7 and 14 DAS and that of *E. colona* by 14 and 21 DAS (Table 3).

**Table 3.** Comparative lengths of roots (mm) of *E. colona*, *E. crus-galli* and rice cultivars ‘Khao Hlan On’ and ‘IR42’ at different flooding depths and timing. Seeds were sown in soil and flooded to depths of 5, 10 and 20 mm. Controls were sown under aerobic conditions (0 mm). Flooding was started 0, 2 or 4 DAS and terminated 7, 14 or 21 DAS. Bold numbers with (*) indicate values are significantly different from the control at LSD_0.05_.

Flooding treatment		7 DAS				14 DAS				21 DAS			
Start	Depth (mm)	*E. colona*	*E. crus-galli*	‘IR42’	‘Khao Hlan On’	*E. colona*	*E. crus-galli*	‘IR42’	‘Khao Hlan On’	*E. colona*	*E. crus-galli*	‘IR42’	‘Khao Hlan On’
0 DAS	0	41	57	108	101	84	112	127	137	170	236	159	161
	5	31	60	69*	88*	64*	92*	111*	119*	130*	193*	149	151
	10	27*	48	55*	69*	55*	90*	101*	118*	131*	185*	151	146
	20	27*	48	55*	69*	52*	85*	94*	101*	119*	165*	138	141
		* *	* *	LSD_0.05_	*11*			LSD_0.05_	*12*	* *	* *	LSD_0.05_	*24*
2 DAS	0	43	58	109	112	83	115	133	128	169	237	153	159
	5	35	53	83*	84*	75	98*	113*	121	159	204*	157	151
	10	30*	45	67*	79*	68*	97*	114*	117	146	195*	157	147
	20	27*	47	68*	66*	59*	92*	106*	111*	124*	184*	141	145
		* *	* *	LSD_0.05_	*11*			LSD_0.05_	*12*	* *	* *	LSD_0.05_	*24*
4 DAS	0	40	59	114	108	81	108	127	141	178	225	158	164
	5	39	57	95*	85*	77	110	123	127*	158*	222	155	147
	10	35	52	79*	75*	75	108	118	129*	148*	218	156	148
	20	34	54	92*	85*	68*	102	110*	114*	141*	205	161	148
		* *	* *	LSD_0.05_	*9*			LSD_0.05_	*9*	* *	* *	LSD_0.05_	*25*

#### Effects of early deep flooding

**Figure 2.**
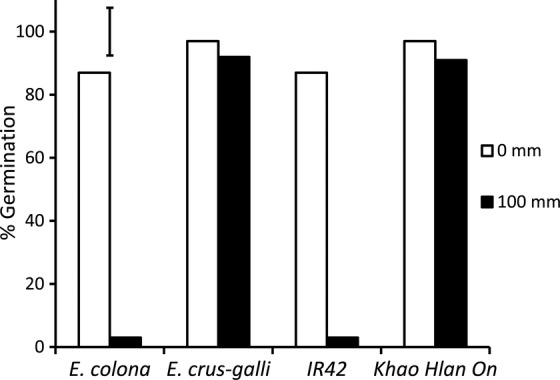
Percent germination of *E. crus-galli*, *E. colona*, ‘Khao Hlan On’ and ‘IR42’ submerged in 0 and 100 mm of water immediately after seeding. Vertical bars indicate l.s.d. at *P* < 0.05.

**Figure 3.**
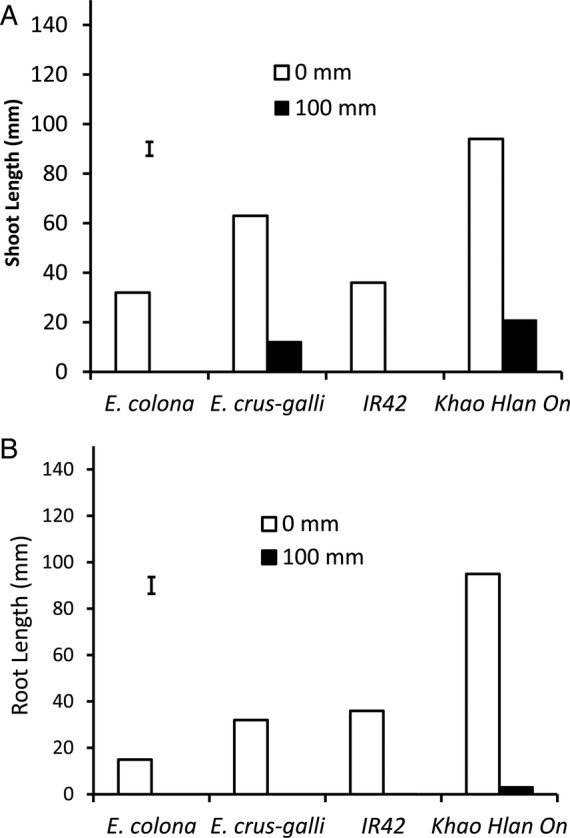
Shoot length (A) and root length (B) of *E. colona*, *E. crus-galli*, ‘IR42’ and ‘Khao Hlan On’ germinated under 100 mm of water. Controls were kept aerobic (0 mm flooding). Data were taken at 7 DAS and vertical bars indicate l.s.d. at *P* < 0.05.

Flooding with 100 mm of water immediately after sowing reduced germination of the sensitive genotypes in both rice and barnyard grass. Germination in *E. colona* and ‘IR42’ was reduced to 3 % compared with controls, and neither radicles nor coleoptiles developed further (Fig. 2). Conversely, germination of the tolerant *E. crus-galli* and rice cultivar ‘Khao Hlan On’ approached ∼91 %. Subsequent growth, however, was greatly reduced. Shoot length was shortened by 80 % and root length by ∼99 % in ‘Khao Hlan On’ when compared with the aerobic controls (Fig. 3) at 7 DAS. Coleoptiles that emerged were thin and devoid of chlorophyll, but by 14 DAS, both shoot and root developed further, though their length was reduced to ∼50 % in the tolerant genotypes. Sensitive genotypes did not develop further even after 14 DAS.

### Delayed deeper flooding

**Figure 4.**
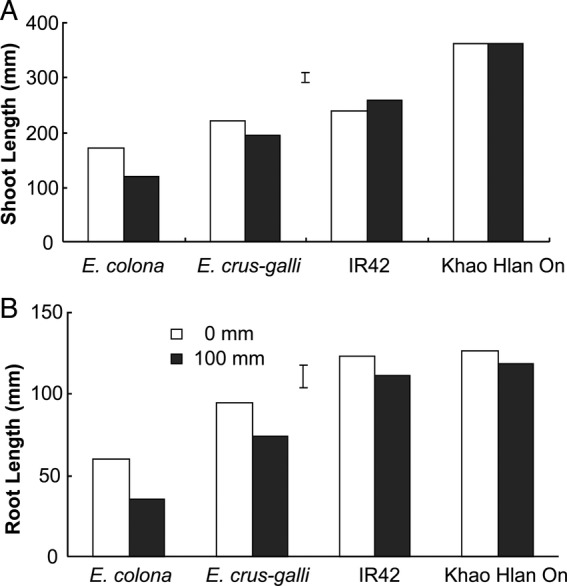
Shoot length (A) and root length (B) of *E. colona*, *E. crus-galli*, ‘IR42’ and ‘Khao Hlan On’ germinated under aerobic conditions for 3 days followed by flooding under 100 mm of water. Controls were kept aerobic (0 mm flooding). Data were taken at 7 DAS and vertical bars indicate l.s.d. at *P* < 0.05.

Delayed flooding of 100 mm applied at 3 DAS did not affect shoot elongation in rice and caused only ∼10 % reduction in root length (Fig. 4A and B). On the other hand, both *E. crus-galli* and *E. colona* genotypes were affected by flooding starting at 3 DAS. Even after 11 days of flooding (14 DAS), shoot lengths of *E. colona* were reduced by an average of 20 % and root lengths of both grasses were reduced by an average of 30 % **[see Supporting Information]**. By 21 DAS however, shoot lengths of both grasses were similar to those of their respective aerobic controls, and similar to those of ‘IR42’. In contrast, root growth of the barnyard grasses remained suppressed under flooded conditions **[see Supporting Information]**.

### Early deep flooding effects on seed carbohydrate utilization in rice and barnyard grasses

**Figure 5.**
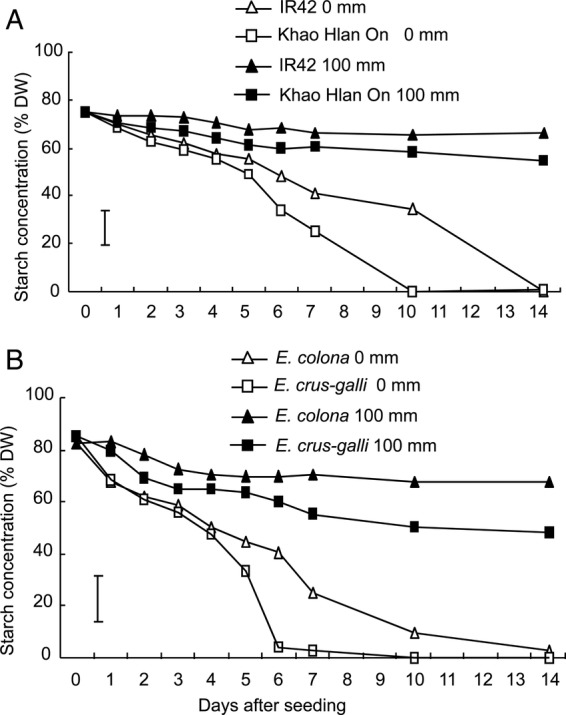
Starch concentration (% DW) of (A) ‘Khao Hlan On’ and ‘IR42’ and (B) *E. crus-galli* and *E. colona* from 0 to 14 days after sowing under aerobic and submerged (100 mm) conditions. Vertical bars indicate l.s.d. at *P* < 0.05.

Starch concentration in germinating seeds of rice and barnyard grasses followed similar trends for either tolerant or sensitive genotypes (Fig. 5A and B). Barnyard grasses had higher starch concentrations in their seeds before germination and degraded them faster. In aerobic soil (0 mm water depth), starch concentration of the tolerant genotypes *E. crus-galli* and ‘Khao Hlan On’ depleted faster, starting at 4 DAS, while the decrease was more gradual in the sensitive *E. colona* and rice genotype ‘IR42’ (Fig. 5A and B). Under 100 mm flooding, starch concentration remained relatively steady in all four genotypes with a gradual decline over the 14 days of sampling. However, the reduction was slightly greater in the tolerant genotypes of both rice and barnyard grass.

**Figure 6.**
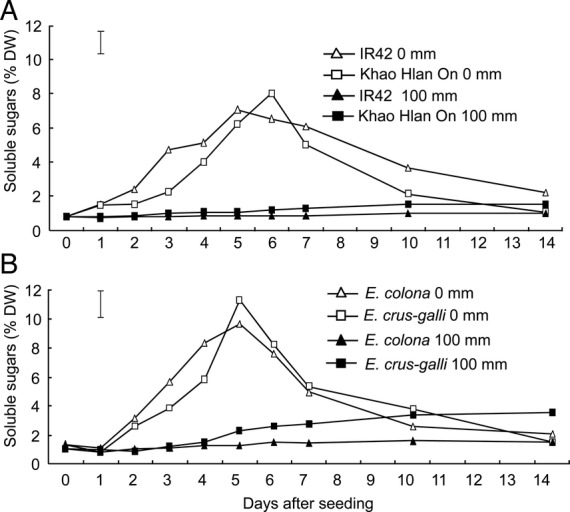
Soluble sugar concentration (% DW) of (A) ‘Khao Hlan On’ and ‘R42’ and (B) *E. crus-galli* and *E. colona* from 0 to 14 days after sowing under aerobic (0 mm) and flooded (100 mm) conditions. Vertical bars indicate l.s.d. at *P* < 0.05.

Soluble sugar concentration in germinating seeds also followed similar patterns in rice and barnyard grass, for both tolerant and sensitive genotypes (Fig. 6A and B). Overall, the barnyard grass maintained higher soluble sugar concentrations than rice. Under aerobic conditions, soluble sugar concentration increased with time and reached a peak at 5–6 DAS, then decreased gradually thereafter (Fig. 6A and B). When submerged under 100 mm of water, the total soluble sugar concentration remained low and largely unchanged in all four genotypes, though the concentration in seeds of the tolerant barnyard grass and rice genotypes was slightly higher but the difference was not significant.

### Activities of enzymes associated with anaerobic respiration

**Figure 7.**
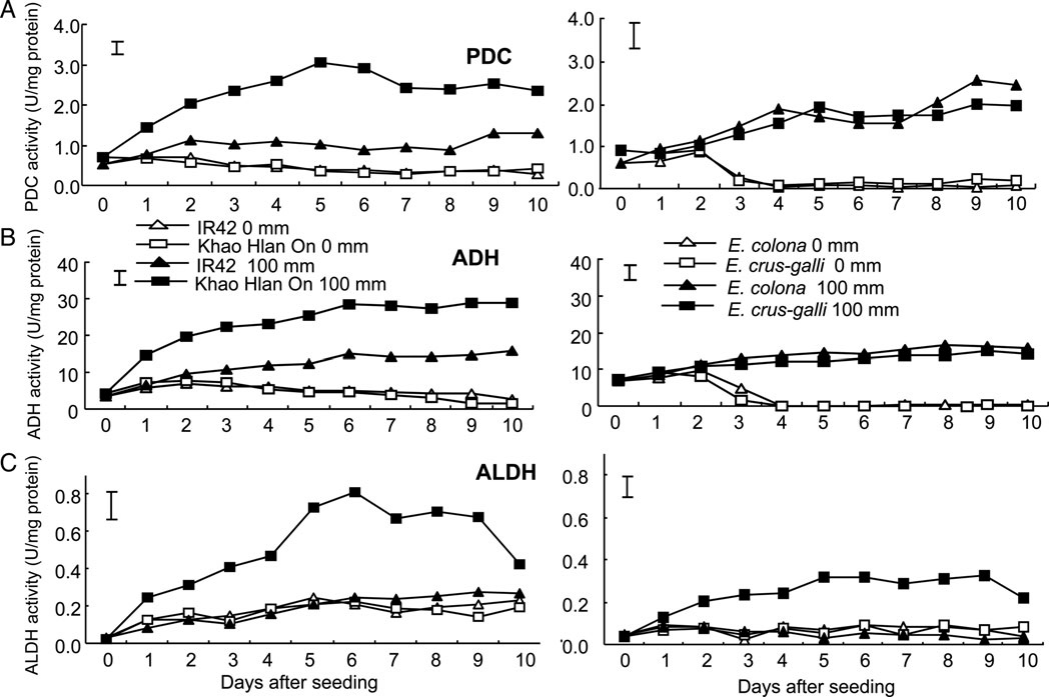
Activities of PDC (A), ADH (B) and ALDH (C) enzymes during germination under aerobic (0 mm) and flooded (100 mm) conditions. The graphs on the left represent activities in ‘Khao Hlan On’ and ‘IR42’ and the graphs on the right represent *E. crus-galli* and *E. colona*. Vertical bars indicate l.s.d. at *P* < 0.05.

Activities of PDC, ADH and ALDH were monitored for both rice and barnyard grass species under aerobic conditions and under early flooding with 100 mm of water depth (Fig. 7). Under aerobic conditions, PDC activity remains low and steady in both rice cultivars but increased substantially when flooded. Pyruvate decarboxylase activity in ‘Kho Hlan On’ increased at a much higher rate, peaking at 5 DAS when flooded. It rose from 0.5 to 3 U mg^−1^ total protein, which is ∼3-fold higher than that of the sensitive ‘IR42’ at the same time. Under aerobic conditions, PDC activity in barnyard grass slightly increased to 0.8 U mg^−1^ total protein during the first 2 DAS, then decreased to close to 0 from 3 to 10 DAS (Fig. 7A). However, when flooded with 100 mm, PDC activity in the two barnyard grasses increased gradually, with similar trends in both species. The activities increased from 0.8 to more than 2.5 U mg^−1^ total protein after 10 DAS (Fig. 7A).

Activities of ADH followed more or less similar patterns to PDC under both aerobic and flooded conditions in both species (Fig. 7B). Under flooded conditions, ADH activity in ‘Khao Hlan On’ increased at a much higher rate, rising from 1 to 30 U mg^−1^ total protein, which is ∼3-fold higher than that of the sensitive ‘IR42’. Barnyard grass germinated in aerobic soil had low ADH activities (Fig. 7B). The activity slightly increased during the first 2 DAS, then decreased to close to 0 from 3 to 10 DAS. When flooded with 100 mm of water, ADH activity increased gradually but similarly slowly in the two barnyard grasses.

Activity of ALDH slowly increased; from close to 0 to 0.2 U mg^−1^ total protein in rice germinated in aerobic conditions for the 10-day period following sowing (Fig. 7C). However, the enzyme activity did not follow the same trend as PDC and ADH when rice was flooded with 100 mm of water. Activity of ALDH in ‘Khao Hlan On’ increased faster and reached a peak at ∼6 DAS (0.8 U mg^−1^ total protein); then declined progressively until 10 DAS, while that of ‘IR42’ remained similar to that under aerobic conditions. The trend was similar in barnyard grass; ALDH remaining steady at ∼0.05 U mg^−1^ total protein both under aerobic conditions and also in the sensitive *E. colona* when flooded. However, ALDH activity in *E. crus-galli* increased significantly under flooding, though the increase was less marked than in ‘Khao Hlan On’. Clearly, ALDH activities increased substantially only in the tolerant genotypes of rice (Khao Hlan On) and barnyard grass (*E. crus-galli*) by about 3- to 4-fold compared with that of the sensitive genotypes ‘IR42’ and *E. colona* under aerobic conditions, with no changes in the sensitive genotypes when flooded. The induction of ALDH under flooded conditions was greater in the tolerant rice genotype than in the tolerant *E. crus-galli*.

### Immunoblot analyses of ALDH2

**Figure 8.**
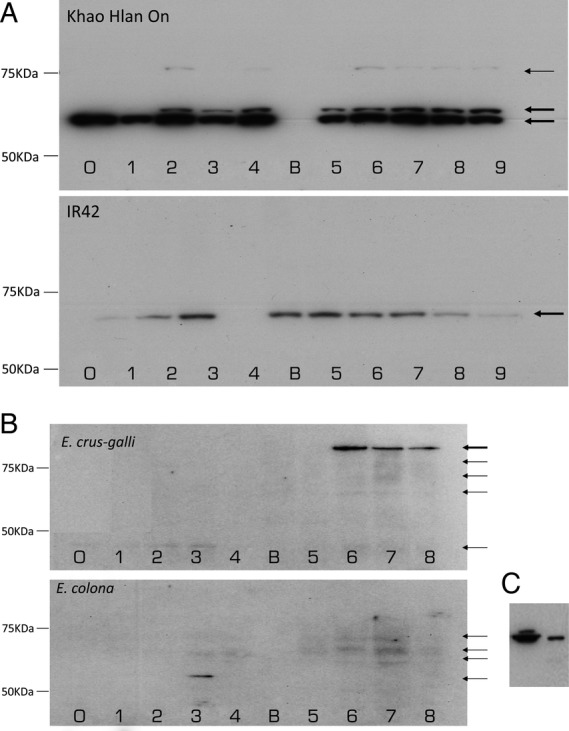
Immunoblot raised against ALDH2 in (A) ‘Khao Hlan On’ and 'IR42′ and (B) *E. crus-galli* and *E. colona*. Samples were sown in the soil flooded with 100 mm of water and harvested daily for 9 days for rice and 8 days for weeds. Black arrows indicate the bands detected in each genotype for ALDH2 proteins. (C) Immunoblot raised against ALDH2 in ‘Khao Hlan On’ (left) and ‘IR42’ (right).

Immunoblotting against ALDH2 was carried out using samples from all genotypes flooded at 100 mm depth starting at Day 0 (Fig. 8). The blot for ‘Khao Hlan On’ and ‘IR42’ (Fig. 8A) showed three and one band, respectively. In ‘Khao Hlan On’, ALDH2b protein was present for all samples from Days 0–9, while ALDH2a protein was detected starting at Day 2 after sowing (Fig. 8A). For ‘IR42’, ALDH2b protein was also detected starting at Day 0 with its expression increased until Day 5, then decreased progressively towards Day 9 (Fig. 8A and C). However, the amount of protein detected by western blot was comparatively lower in ‘IR42’. In barnyard grass, various bands were detected at different molecular weights (Fig. 8B). The tolerant *E. crus-galli* showed a clear banding pattern at a molecular weight of ∼100 KDa, starting at Day 6. A lower band was also detected in *E. crus-galli* starting at Day 0 until Day 8. Three bands were identified in *E. colona*, at a molecular weight of ∼75 KDa. A lower band was also identified at Day 3 only, at a similar molecular weight as ALDH2b in rice.

## Discussion

One of the major constraints of direct-seeded rice is that both the crop and weeds emerge together. However, controlled flooding can be effectively used for weed management by exploiting differential responses in growth and metabolic responses of rice and weeds to flooding. In flooded soils, low oxygen causes germinating seeds to shift from aerobic respiration to the less efficient but more robust anaerobic fermentation and promote a series of biochemical activities to counter the effect of hypoxic conditions (Rumpho and Kennedy 1981, 1983*a*, *b*; Vartapetian *et al.* 2003; Ismail *et al.* 2009; Magneschi and Perata 2009). Once shoots have emerged above the floodwater, the leaves can switch to aerobic respiration.

### Effect of flooding depth and timing on germination and early growth of rice and barnyard grass

Past studies on the tolerance of flooding of the two contrasting rice and barnyard grass genotypes brought new knowledge concerning the efficiency of different flooding regimes to suppress weed growth without affecting rice (Chauhan and Johnson 2011; Ismail *et al.* 2012). However, identifying rice varieties capable of germination under water is essential before effective use can be made of flooding for suppressing weeds that germinate with the rice. Recently, considerable variation in tolerance of flooding during germination was observed in rice and several tolerant landraces were identified and characterized (Ismail *et al.* 2009, 2012; Angaji *et al.* 2010).

In this study, we observed that flooding at different intervals after seeding (0, 2 and 4 DAS) and depths (5–20 mm) did not jeopardize the germination of either rice genotypes or the two barnyard grasses (Fig. 1). However, early flooding with 5–20 mm of water immediately after seeding was more effective in partially suppressing the subsequent growth of barnyard grass than when flooding was imposed at 2 or 4 days later, especially for the sensitive *E. colona*. The two rice cultivars recovered after 3 weeks (Tables 1 and 2). These observations suggest that barnyard grass is more sensitive to early flooding than rice, especially when tolerant rice genotypes like ‘Khao Hlan On’ are used. Both tolerant and sensitive barnyard grasses germinated under shallower flooding (0–20 mm) but *E. colona* showed greater reduction in shoot and root growth, especially under earlier floods (Tables 1 and 2). Mujer *et al.* (1993) also observed variation in tolerance of flooding among *Echinochloa* species. These results thus confirm the relatively higher tolerance of *E. crus-galli* and ‘Khao Hlan On’ of flooding during germination and early growth.

Seedlings of all four genotypes flooded under 100 mm of water (starting at sowing and continued for 7 days) showed a reduction in germination and growth of roots and shoots (leaves); however, the reduction was much greater in the sensitive *E. colona* and rice ‘IR42’ genotype (Fig. 3). Shoot growth of tolerant *E. crus-galli* was more severely reduced compared with that of ‘Khao Hlan On’, whereas root growth ceased completely in the sensitive rice genotype and in both barnyard grasses under these conditions (Fig. 3). This result indicates that tolerant rice genotypes are likely to gain a competitive advantage over barnyard grass during germination and early growth when flooded at the time of seeding. On the other hand, flooding to 100 mm at a later stage (3 DAS) did not suppress the growth of either rice or tolerant *E. crus-galli*, indicating that delayed flooding is ineffective compared with flooding at sowing (Fig. 4). Clearly however, early flooding will be effective when combined with the use of a tolerant rice genotype such as ‘Khao Hlan On’. Additional studies on the responses of other weed species to flooding are desirable. One such species might well be *Ludwigia hyssopifolia* (Chauhan *et al.* 2011), which is becoming increasingly prevalent in direct-seeded rice. Broadening the work in this way could help fine-tune water and crop management practices to control these weeds in direct seeding rice systems.

### Early deep flooding effects on seed carbohydrate breakdown and utilization in rice and barnyard grass

Since flooding at 100 mm from Day 0 seemed to be the most effective strategy for weed suppression in direct-seeded rice, further analyses were performed using these conditions alone. Starch concentration in barnyard grass and rice seeds decreased when germinating under aerobic conditions (Fig. 5). However, soluble sugar concentration increased during the first 5 days after flooding and decreased afterwards (Fig. 6). This observation is consistent with the breakdown of starch into sugars and then the utilization of sugars in aerobic respiration. Murata *et al.* (1968) and Nomura *et al.* (1969) reported an increase in sugar concentration in seeds of aerobically grown rice in the first 3 to 4 days. We also observed more rapid starch utilization in *E. crus-galli*, which might contribute to its competitiveness against rice. Kim and Moody (1989) noted that, in early growth, barnyard grass had a 4-day ‘head start’ over rice.

When rice and barnyard grass germinated and grew under flooded conditions, there was little change in starch concentration and little or no increases in soluble sugar concentration. This reflected a substantial reduction in the ability of these genotypes to mobilize starch into soluble sugars while submerged. The slow increase (albeit statistically not significant) in starch breakdown and sugar concentration over time in the tolerant rice and barnyard grass species is possibly sufficient for slower carbohydrate metabolism in both *E. crus-galli* and ‘Khao Hlan On’. Moreover, the lower rate of carbohydrate catabolism agreed with the slower rate of germination and emergence observed in the flooded treatments. Some studies have previously reported carbohydrate metabolism occurring in *E. crus-galli* under anoxia (Kennedy *et al.* 1980; Vanderzee and Kennedy 1981). The tolerant rice genotypes degraded starch at a higher rate under flooded conditions compared with intolerant genotypes (Ismail *et al.* 2009). Kato-Noguchi *et al.* (2008) suggested that this ability to degrade starch under low oxygen stress confers anoxia tolerance in rice. The failure of flood-intolerant crops, such as wheat, to germinate under anoxia has been correlated with their inability or decreased ability to degrade starch under these conditions (Perata *et al.* 1997). An increased sugar concentration, resulting from starch degradation by amylase or sucrose synthase, has been observed in plants tolerant of anaerobic conditions (e.g. *Cyperus rotundus* L. [Peña-Fronteras *et al.* 2009]; rice [Guglielminetti *et al.* 1995; Ismail *et al.* 2009]).

### PDC, ADH and aldehyde dehydrogenase activities under aerobic and 100 mm flooding conditions

In this study, we found that both rice and *Echinochloa* spp. have higher PDC and ADH activities when germinating under flooded conditions compared with aerobic soil (Fig. 7). This indicates that enzymes of ethanol fermentation are induced under hypoxic conditions in both rice and barnyard grass. In rice, ‘Khao Hlan On’ showed about twice the activities of PDC and ADH of that of the sensitive ‘IR42’, which might partially account for the greater flood tolerance of this genotype. On the other hand, similar increases in ADH and PDC activities were observed in the two barnyard grasses in flooded soil. This suggests that variation in the induction of this pathway might not be associated with variation in flooding tolerance in these species. A possible cause would be the relatively small pool of total carbohydrate content in the small seeds of these weeds compared with rice seeds. Earlier findings showed that both PDC and ADH activities were induced under hypoxia in rice (Ismail *et al.* 2009) and *E. crus-galli* (Fukao *et al.* 2003). Increases in PDC and ADH activities have been associated with tolerance in various species. Valdez (1995) and Ismail *et al.* (2009) reported higher PDC and ADH activities in tolerant rice genotypes when compared with sensitive ones; Peña-Fronteras *et al.* (2009) and Fuentes *et al.* (2010) showed similar results in flood-tolerant lowland ecotypes of *C. rotundus;* and this was also reported in other plants such as maize (Schwartz 1969) and *A. thaliana* (Dolferus *et al.* 2003).

The lower activities of ADH and PDC in barnyard grasses germinating under aerobic conditions and their further reduction observed 3 days after sowing suggest that these enzymes are probably not necessary for barnyard grass seeds germinating in air. In contrast, these enzymes were active in both rice genotypes in aerobic soil, indicating that some degree of anaerobic respiration is taking place, possibly because of their relatively large seed size and the possibility of hypoxic pockets existing within the seed during germination (Colmer *et al.* 2014). This supports a more general view that suboptimal oxygen conditions occur in rice and other cereal seeds during germination (Zabalza *et al.* 2009). Seeds of the two barnyard grasses are very much smaller (1000 grain weight of 1.5 g for *E. colona* and 2.9 g for *E. crus-galli*) compared with the two rice species (1000 grain weight of 23 g for ‘Khao Hlan On’ and 18 g for ‘IR42’).

Aldehyde dehydrogenase activity increased substantially in the tolerant rice ‘Khao Hlan On’ and tolerant *E. crus-galli* when grown in flooded soil. However, the enzyme did not show high activity in either of the rice cultivars or barnyard grass species when they were grown in aerobic soil. This observation suggests that this pathway is similarly important for tolerance of hypoxic conditions during germination in both rice and barnyard grass. Increased ALDH activity in *E. crus-galli* var. *formosensis* (Fukao *et al.* 2003) and in some rice cultivars (Nakazono *et al.* 2000; Magneschi and Perata 2009) has been suggested as one of the reasons for tolerance of anaerobic conditions. This could possibly be mediated through detoxification of excess acetaldehyde generated during anaerobic fermentation (Miro and Ismail 2013).

Metabolic coping strategies of rice and barnyard grass may not be identical. However, there are similar patterns for changes in starch, soluble sugar and ALDH activities in rice and barnyard grasses. Both barnyard grasses have small seeds that store limited amounts of carbohydrates for germination. Indeed, their germination rates are much faster than those of rice, which has larger seeds and a correspondingly larger carbohydrate store. This correlates with starch and soluble sugar data (Figs 5 and 6), where barnyard grass shows faster hydrolyses of reserves than rice. A faster metabolism may be an advantage in delayed and shallow flooding but not in early and deeper flooding.

### Immunoblot assays for ALDH2 in rice and barnyard grass under 100 mm flooding

The increase in ALDH activity was seen only in tolerant rice cultivar ‘Khao Hlan On’ and tolerant barnyard grass *E. crus-galli*. Western blots using antibodies raised against ALDH2 from rice and *Arabidopsis thaliana* were carried out to assess the protein patterns of this family in tolerant and sensitive genotypes. Aldehyde dehydrogenases belong to a well-characterized family of proteins previously studied in relation to submergence (Nakazono *et al.* 2000; Tsuji *et al.* 2003*b*). Of the different ALDH family proteins, ALDH2 has been identified as differentially expressed and translated in contrasting rice genotypes via proteomic analyses under anaerobic conditions (Sadiq *et al.* 2011). Of the two enzymes known for ALDH2, we found that both ALDH2a and ALDH2b are present in higher concentrations in the tolerant rice genotype, showing constant levels of translation. The sensitive ‘IR42’, on the other hand, showed low levels of ALDH2b under submergence. The proteins were identified based on their molecular weight. ALDH2b was the lowest band in ‘Khao Hlan On’ and the only band in ‘IR42’ (Fig. 8C). ALDH2a was the middle band in ‘Khao Hlan On’ (Tsuji *et al.* 2003*a*; Kotchoni *et al.* 2010). This observation is in agreement with those of Tsuji *et al.* (2003*b*) and Sadiq *et al.* (2011), who also found low levels of ALDH2b protein. In our study, however, ALDH2b protein was present in high concentrations in tolerant rice ‘Khao Hlan On’ and it did not decrease over time. On the other hand, our results on ALDH2a are in agreement with previous findings, suggesting low protein levels in both rice varieties (Tsuji *et al.* 2003*b*; Sadiq *et al.* 2011). ALDH2a was undetectable by immunoblot in ‘IR42’. Genomic clones of both ALDH2a and ALDH2b were sequenced and compared but were found to be identical. Similar observations have been made for the CDS sequences of both genes (data not shown). Through gene network models, future work will focus on transcription factors, kinases and other pathway-related genes responsible for differential expression of ALDH. These results will shed light on the complex mechanisms involved in the regulation of genes associated with anaerobic metabolism during germination.

The immunoblot raised against ALDH2 did not show a very clear banding pattern in barnyard grass. One reason is probably the heterologous nature of the antibody. Since ALDH2 is a highly conserved protein across species, the blotting results can be attributed to ALDH2 in the barnyard grass, especially in *E. crus-galli* on Days 6, 7 and 8. However, it cannot be confirmed with actual data whether ALDH2a or ALDH2b or another similar ALDH were detected. A comparison of the blots obtained from tolerant rice and barnyard grass seems to indicate that one of the ALDH2 forms has been present since the beginning of germination in both ‘Khao Hlan On’ and *E. crus-galli*, and that the protein can be detected throughout until Day 8 (Fig. 8). However, any possible relationship between high levels of ALDH2 and tolerance needs to be explored further.

## Conclusions

One of the major problems of using flooding of rice fields as a weed control measure is the lack of information on differential responses of rice and weeds to this practice during the first 2 weeks of crop establishment. Unravelling these responses will help design better management options that reduce weed infestation while inflicting minimal damage to the rice. Neither shallow nor delayed flooding was sufficient to suppress the growth of barnyard grass species used in this study; however, flooding with 100 mm of water immediately after sowing was effective with much less inhibitory effect on the growth of the tolerant rice genotype ‘Khao Hlan On’. Flooding of 20–40 mm applied under field conditions was recently reported to be effective in suppressing intolerant weeds such as *E. colona* (Chauhan 2012). Metabolic differences between tolerant and sensitive rice genotypes and barnyard grass were also investigated. Anaerobic respiration was induced similarly in both flood-tolerant and flood-sensitive barnyard grasses but this induction was substantially enhanced in the tolerant rice ‘Khao Hlan On’ compared with flood-sensitive ‘IR42’. Aldehyde dehydrogenase activity was enhanced by flooding only in tolerant *E. crus-galli* and tolerant rice ‘Khao Hlan On’ but not in sensitive *E. colona* and ‘IR42’, suggesting a causal association with tolerance in both barnyard grass and rice. Further studies are needed to unravel the control mechanisms that mediate the upregulation of ALDH in tolerant rice and barnyard grass to establish its role in adaptation to flooding. These findings will ultimately help to develop flooding and other weed management strategies to control weeds effectively while minimizing damage to the rice crop itself.

## Sources of Funding

The research was partly funded through the Philippine Council for Advanced Science and Technology Research and Development, the Irrigated Rice Research Consortium of the International Rice Research Institute and the Bill and Melinda Gates Foundation.

## Contributions by the Authors

L.P.E. undertook the physiology work and enzymatic activity analyses. B.M. took part in the enzyme activity assays and handled the western blot analyses. All authors contributed to the planning of the research and the preparation of the manuscript.

## Conflicts of Interest Statement

None declared.

## Supporting Information

The following Supporting Information is available in the online version of this article –

**Figure S1.** Seedlings of barnyard grass and rice after germination under aerobic conditions (left) and under 20 mm of flooding starting at sowing (right): (1) *E. colona*, (2) *E. crus-galli*, (3) ‘IR42’ and (4) ‘Khao Hlan On’. Pictures were taken at 7, 14 and 21 DAS. The vertical white bar represents 10 cm. Root length was clearly reduced under submerged conditions in all genotypes. Seedlings of both echinochloa species showed reduced growth, especially at 7 DAS; however, by 21 DAS, surviving seedlings of both rice and barnyard grass seemed to have recovered.

**Figure S2.** Fourteen-day-old seedlings of *E. colona* and *E. crus-galli*, ‘IR42’ and ‘Khao Hlan On’ under (A) aerobic and (F) flooding with 100 mm of water after germinating aerobically for 3 days. The vertical white bar represents 10 cm. All genotypes seemed to partially recover after 14 days, except for *E. colona,* which showed reduced growth.

Additional Information
