# Supplementary material for: Differences in responses to flooding by germinating seeds of two contrasting rice cultivars and two species of economically important grass weeds
Source: AoB Plants. 2014 Oct 20;6:plu064. doi: 10.1093/aobpla/plu064 (PMC4243074; doi:10.1093/aobpla/plu064)
Supplement: Additional Information [file supp_6_plu064_index.html]

Differences in responses to flooding by germinating seeds of two contrasting rice cultivars and two species of economically important grass weeds — Differences in responses to flooding by germinating seeds of two contrasting rice cultivars and two species of economically important grass weeds — Additional Information 

# Differences in responses to flooding by germinating seeds of two contrasting rice cultivars and two species of economically important grass weeds

## Additional Information

Additional Information

**Files in this Data Supplement:**

- Supporting Information - pptx file
