# Supplementary material for: Differences in responses to flooding by germinating seeds of two contrasting rice cultivars and two species of economically important grass weeds
Source: AoB Plants. 2014 Oct 20;6:plu064. doi: 10.1093/aobpla/plu064 (PMC4243074; doi:10.1093/aobpla/plu064)
Supplement: Additional Information [file supp_plu064_plu064supp.pptx]

## Slide 1
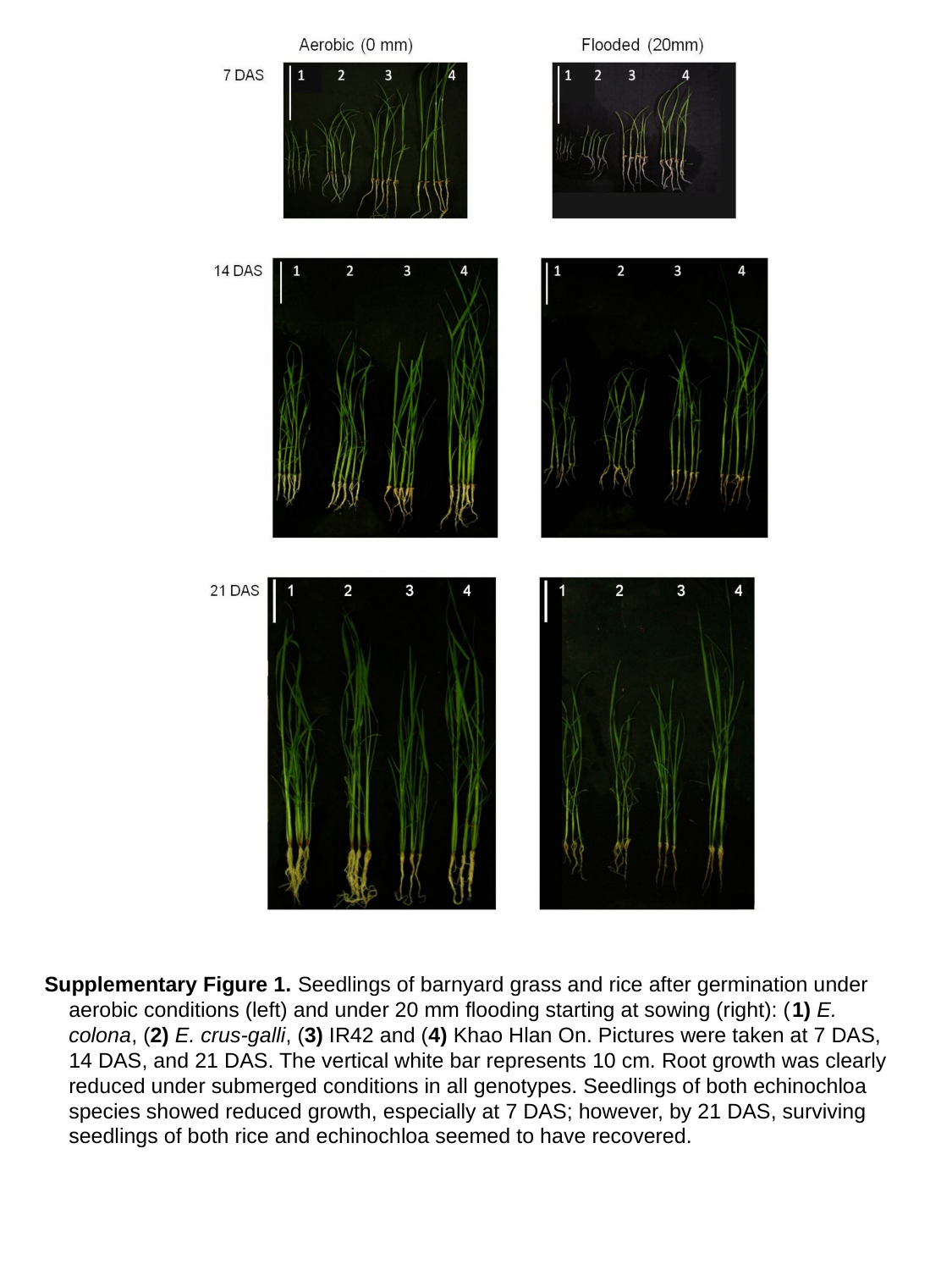

Supplementary Figure 1. Seedlings of barnyard grass and rice after germination under aerobic conditions (left) and under 20 mm flooding starting at sowing (right): (1) E. colona, (2) E. crus-galli, (3) IR42 and (4) Khao Hlan On. Pictures were taken at 7 DAS, 14 DAS, and 21 DAS. The vertical white bar represents 10 cm. Root growth was clearly reduced under submerged conditions in all genotypes. Seedlings of both echinochloa species showed reduced growth, especially at 7 DAS; however, by 21 DAS, surviving seedlings of both rice and echinochloa seemed to have recovered.

## Slide 2
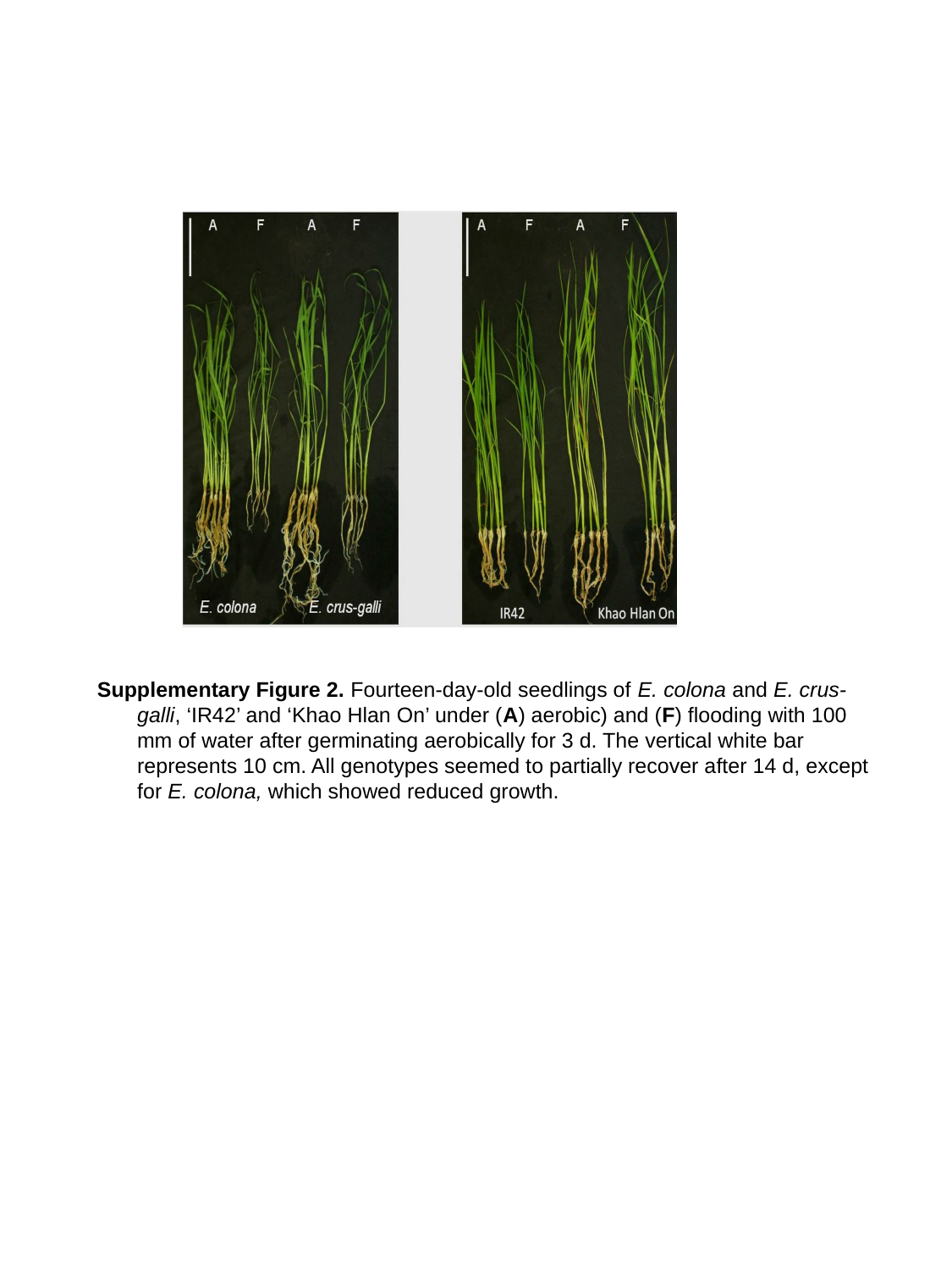

Supplementary Figure 2. Fourteen-day-old seedlings of E. colona and E. crus-galli, ‘IR42’ and ‘Khao Hlan On’ under (A) aerobic) and (F) flooding with 100 mm of water after germinating aerobically for 3 d. The vertical white bar represents 10 cm. All genotypes seemed to partially recover after 14 d, except for E. colona, which showed reduced growth.
